# Acute Bacterial Prostatitis in a 12-Year-Old Boy Without Any Underlying Disease

**DOI:** 10.7759/cureus.60900

**Published:** 2024-05-23

**Authors:** Kaoru Kinoshita, Michio Suzuki, Masaki Koketsu, Tatsuya Fukasawa, Tetsuo Kubota

**Affiliations:** 1 Pediatrics, Anjo Kosei Hospital, Aichi, JPN

**Keywords:** contrast-enhanced computed tomography, corynebacterium pyruviciproducens, prostatitis abscess, child, acute bacterial prostatitis

## Abstract

Acute bacterial prostatitis (ABP) is a common disease in adults but uncommon in children. Here, we report the case of a pediatric patient without any underlying disease who was diagnosed with ABP while trying to determine the cause of fever refractory to antimicrobial therapy. A previously healthy 12-year-old boy presented with a 13-day history of fever and malaise despite initial antimicrobial treatment. Further tests revealed pyuria and enlarged prostate with possible abscesses, which led to the diagnosis of ABP based on a contrast-enhanced computed tomography (CT) scan. Although initial urine cultures were negative, *Corynebacterium pyruviciproducens* was detected in subsequent cultures. Antimicrobial therapy for 10 weeks led to improvement without relapse. This case demonstrates that ABP can cause fever in children. Moreover, it shows that contrast-enhanced CT imaging can help identify the cause of fever and that administration of antimicrobials before adequate investigations can confound the diagnosis and complicate the treatment.

## Introduction

Acute bacterial prostatitis (ABP) is an acute infection of the prostate gland. ABP is caused by well-recognized uropathogenic bacteria and leads to acute symptoms of urinary tract infection, such as urinary frequency and dysuria, and may also result in fever and other systemic symptoms [1]. Although prostatitis is common in middle-aged men in clinical settings, only few cases have been reported in children, and all of these cases were caused by some underlying disease [2-7].

Herein, we report the case of a 12-year-old boy without any underlying disease who had persistent fever and was diagnosed with ABP using contrast-enhanced computed tomography (CT). Urine culture revealed insufficient amounts of *Corynebacterium pyruviciproducens* and thus, the causative pathogen could not be identified. Although ABP with small abscesses was suspected, the patient was cured only via long-term antimicrobial treatment.

## Case presentation

**Figure 1:**
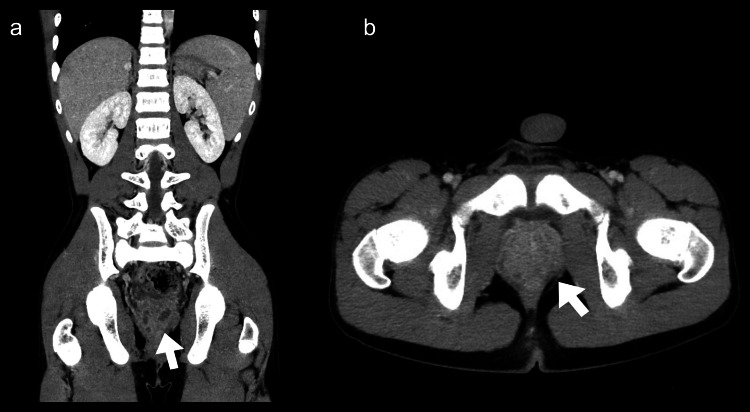
Contrast-enhanced computed tomography images of the patient (a) Coronal view, (b) axial view. The white arrows indicate diffuse enlargement of the prostate and numerous areas of poor contrast within the prostate.

A 12-year-old boy presented to our hospital with a 13-day history of fever and malaise. He denied any previous medical history, sexual history, or recent trauma history and had never masturbated or ejaculated. He was prescribed clarithromycin, cefditoren pivoxil, and fosfomycin for five, four, and four days, respectively, but his fever persisted. The patient’s physical examination was normal, and his body temperature was 37.3°C. Blood tests showed a white blood cell count of 17,100/μL and a C-reactive protein (CRP) of 3.62 mg/dL (0.00-0.14 mg/dL), and urine sediment showed pyuria with 50-99 white blood cells/high power field. Blood, urine, stool, and pharyngeal samples were collected, and the patient was admitted to the hospital for observation. Following admission, fever (up to 38°C) continued until the third day of admission. A contrast-enhanced CT scan of the region from the neck to the pelvis was performed to determine the origin of the fever and indicated diffuse enlargement of the prostate and numerous areas of poor contrast within it (Figure 1), which led to the diagnosis of ABP.

**Figure 2:**
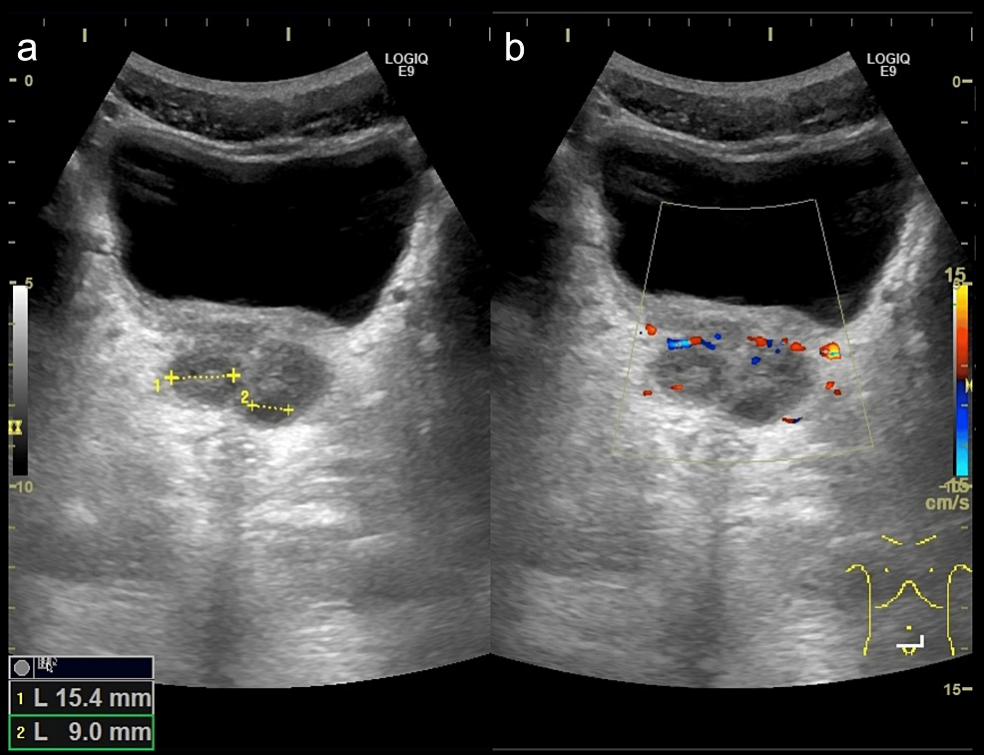
Ultrasonography images Ultrasonography revealed an enlarged prostate and multiple small cysts, which were 15.4 mm and 9 mm in diameter (a), and increased color flow (b).

Furthermore, ultrasonography revealed an enlarged prostate, increased color flow, and multiple small cysts, which were possibly abscesses (Figure 2).

Intravenous cefotaxime and oral azithromycin were administered, although there was no history of sexual intercourse as per the interview. Only cefotaxime was continued after the urinalysis of polymerase chain reaction was negative for *Neisseria gonorrhoeae* and *Chlamydia* *trachomatis*. Although pyuria improved gradually, low-grade fever persisted for a while despite antimicrobial therapy. Cultures of urine and blood samples collected on the first day of hospitalization did not detect any microorganisms. However, urine culture on the third day showed *C. pyruviciproducens* at a density of 10^2^ colony-forming units (CFU) /mL, although the presence of bacteria was not confirmed via Gram staining. Antimicrobial susceptibility was determined using the disk method, which indicated that the organism was susceptible to the antibiotics penicillin G, cefotaxime, meropenem, and vancomycin; however, it was resistant to ciprofloxacin. Owing to antibiotic susceptibility and drug translocation to the prostate and considering other Enterobacterales, intravenous ampicillin and trimethoprim-sulfamethoxazole were administered from the 16th day of hospitalization. Subsequently, the therapy was switched to oral amoxicillin and trimethoprim-sulfamethoxazole on the 22nd day, and the patient was discharged. From the second day after discharge, as trimethoprim-sulfamethoxazole resulted in a drug rash, amoxicillin alone was continued. Additional antimicrobial treatment was administered every two weeks after discharge, and blood and urine tests and ultrasonography were performed. CRP was reduced to 0.09 mg/dL, prostate enlargement was alleviated, and small cysts disappeared after a total of 10 weeks of antimicrobial treatment, and there was no relapse after the treatment ended.

## Discussion

This article reports the case of a previously healthy 12-year-old boy with persistent fever resistant to oral antimicrobials who was diagnosed with ABP using contrast-enhanced CT. On the second day after the discontinuation of antimicrobial treatment, only *C. pyruviciproducens *was detected in the urine culture.

ABP, which is common in adults but uncommon in children, was the cause of the prolonged fever in the presenting case who did not have any underlying disease. In 1999, the National Institutes of Health consensus statement on prostatitis was proposed. According to this statement, the disease is categorized into four broad types: ABP, chronic bacterial prostatitis, chronic prostatitis/chronic pelvic pain syndrome, and asymptomatic inflammatory prostatitis [1]. ABP refers to an acute infection of the prostate gland that causes pelvic pain and urinary tract symptoms, such as dysuria, urinary frequency, and urinary retention. Additionally, systemic symptoms, such as fever, chills, nausea, and malaise, may be noted [1]. In this case, the patient experienced only fever and malaise. The other symptoms could have been masked as he had been administered antimicrobials before the visit. Reflux of infected urine into the ejaculatory and prostatic ducts and an ascending urethral infection from the distal urethra are the major mechanisms of ABP. Occasionally, ABP is caused by direct or lymphatic spread from the rectum or hematogenous spread via bacterial sepsis. The development of prostatitis may be affected by underlying functional or anatomical anomalies that predispose to urogenital infections [8]. However, only a few cases of ABP or prostate abscess (PA) have been reported in the pediatric population. These cases originated from certain underlying diseases, including bacteremia [2-4], chronic granulomatous disease [5], intense undernutrition owing to picky eating in autism [6], abnormal urinary habits, and a history of recurrent urinary tract infection [7]. A bacteremia case occurred following trauma to the lower abdomen [4]. Our patient did not have bacteremia, a compromised immune system, urologic anomalies, or trauma, and is hence an unusual case.

The diagnosis of ABP is usually based on typical signs and symptoms. Tenderness of the prostate is usually perceived during rectal examination [9]. Imaging studies are not essential for the diagnosis. Nonetheless, some studies have observed that ultrasonography is a useful tool to evaluate and monitor the treatment response. Intraprostatic color flow in patients with ABP is greater than that in patients with normal prostate and those with chronic inflammation or infection recovery [10]. In this case, the diagnosis was based on characteristic imaging findings, and rectal examination was not performed owing to its invasiveness. Despite concerns about radiation exposure, particularly in children, contrast-enhanced CT imaging may be beneficial in identifying the cause in unusual cases such as this one.

In the present case, although *C. pyruviciproducens* was detected in the urine culture, the exact causative pathogen could not be determined. *Escherichia coli *is the most common pathogen encountered in ABP. Enterobacterales, such as *Klebsiella* and *Proteus* species, and nonfermenting gram-negative bacilli, such as *Pseudomonas* species and *Enterococcus* species, are the other pathogens involved. *N. gonorrhoeae* and *C. trachomatis* should be considered in sexually active men [11]. In previous reports of ABP or PA involving children, urine culture revealed *Serratia marcescens* [3], *Pseudomonas aeruginosa* [5], *E. coli* [6], and no bacteria [2,4,7]. 54% of the urine cultures of 192 cases of ABP in adults revealed *E. coli*, whereas 34% failed to detect any organism; in the patients with negative urine microbiology, 54% were administered antimicrobials by their general practitioners [12]. In our case, these common pathogens were not detected and only *C. pyruviciproducens* was identified. We consider that the urine culture in the present case implies the absence of other bacteria owing to the administration of antimicrobial agents before admission. *C. pyruviciproducens *was first described in 2010 based on the features of a single strain cultured from a groin abscess [13]. *C. pyruviciproducens *is a gram-positive non spore-forming rod capable of growing aerobically or facultatively anaerobically at 37°C and 42°C. This organism is susceptible to the antibiotics gentamicin, vancomycin, rifampin, linezolid, daptomycin, etc. However, its susceptibilities to beta-lactams, erythromycin, clindamycin, fluoroquinolones, tetracycline, and trimethoprim-sulfamethoxazole are variable. Details such as pathogenicity have not yet been elucidated, but some were detected in urine culture [14]. In the present case, only 10^2^ CFU/mL of *C. pyruviciproducens* was detected in the urine culture, which implies the possibility of contamination with *C. pyruviciproducens.* Although *C. pyruviciproducens* was not confirmed as the causative pathogen, we considered that it could be one of the causative microorganisms and selected antimicrobial agents, assuming *C. pyruviciproducens* and other Enterobacterales.

Antibiotic therapy should be continued for at least two to four weeks although there has been no meta-analysis or randomized controlled trial on antibiotic selection and duration of treatment for ABP. Most febrile cases become afebrile within 36-48 hours after initiating antibiotic therapy. Otherwise, complications with PA should be noted; PA is present in 2%-18% of ABP and requires a prolonged treatment protocol or surgical drainage [8]. In previous reports of ABP or PA involving children, antimicrobials were administered for approximately 2-4 weeks [2,4-7] and drainage was performed in two cases [2,4]. One patient experienced a relapse [5], and one patient died within 36 hours [3]. In the present case, despite antimicrobial therapy, the patient had persistent low-grade fever, and the findings suggested the presence of microabscesses. Therefore, he was treated with antimicrobials for a total of 10 weeks until the CRP levels normalized, prostate enlargement improved, and the cysts disappeared. The duration of treatment in the present study was longer than that reported in previous studies owing to complications due to numerous small abscesses, which were difficult to drain.

## Conclusions

In conclusion, we have reported the case of a 12-year-old boy without any underlying disease who was diagnosed with ABP, and only *C. pyruviciproducens* was detected in urine culture. The causative pathogen was not clearly identified, and complications with many small abscesses were suspected, which made the case difficult to treat. The findings from this case illustrate that ABP can cause fever in children too. In spite of concerns related to radiation exposure, especially in children, contrast-enhanced CT imaging may aid in determining the cause in uncommon cases. Furthermore, it should be remembered that administrating antimicrobials before adequate examinations may lead to unclear causes and refractory treatment.
